# Gradient boosting decision tree becomes more reliable than logistic regression in predicting probability for diabetes with big data

**DOI:** 10.1038/s41598-022-20149-z

**Published:** 2022-10-11

**Authors:** Hiroe Seto, Asuka Oyama, Shuji Kitora, Hiroshi Toki, Ryohei Yamamoto, Jun’ichi Kotoku, Akihiro Haga, Maki Shinzawa, Miyae Yamakawa, Sakiko Fukui, Toshiki Moriyama

**Affiliations:** 1 Health Care Division, Health and Counseling Center, Osaka University, Osaka, 560-0043 Japan; 2 Graduate School of Human Sciences, Osaka University, Osaka, 565-0871 Japan; 3 Research Center for Nuclear Physics, Osaka University, Osaka, 567-0047 Japan; 4 Department of Nephrology, Graduate School of Medicine, Osaka University, Osaka, 565-0871 Japan; 5 Health Promotion and Regulation, Department of Health Promotion Medicine, Osaka University Graduate School of Medicine, Osaka, 565-0871 Japan; 6 Graduate School of Medical Care and Technology, Teikyo University, Tokyo, 173-8605 Japan; 7 Graduate School of Biomedical Sciences, Tokushima University, Tokushima, 770-8503 Japan; 8 Division of Health Sciences, Graduate School of Medicine, Osaka University, Osaka, 565-0871 Japan; 9 Department of Home and Palliative Care Nursing, Graduate School of Health Care Sciences, Tokyo Medical and Dental University, Tokyo, 113-8519 Japan

**Keywords:** Diabetes, Disease prevention, Physical examination, Computational science, Computer science, Information technology

## Abstract

We sought to verify the reliability of machine learning (ML) in developing diabetes prediction models by utilizing big data. To this end, we compared the reliability of gradient boosting decision tree (GBDT) and logistic regression (LR) models using data obtained from the Kokuho-database of the Osaka prefecture, Japan. To develop the models, we focused on 16 predictors from health checkup data from April 2013 to December 2014. A total of 277,651 eligible participants were studied. The prediction models were developed using a light gradient boosting machine (LightGBM), which is an effective GBDT implementation algorithm, and LR. Their reliabilities were measured based on expected calibration error (ECE), negative log-likelihood (Logloss), and reliability diagrams. Similarly, their classification accuracies were measured in the area under the curve (AUC). We further analyzed their reliabilities while changing the sample size for training. Among the 277,651 participants, 15,900 (7978 males and 7922 females) were newly diagnosed with diabetes within 3 years. LightGBM (LR) achieved an ECE of 0.0018 ± 0.00033 (0.0048 ± 0.00058), a Logloss of 0.167 ± 0.00062 (0.172 ± 0.00090), and an AUC of 0.844 ± 0.0025 (0.826 ± 0.0035). From sample size analysis, the reliability of LightGBM became higher than LR when the sample size increased more than $10^4$. Thus, we confirmed that GBDT provides a more reliable model than that of LR in the development of diabetes prediction models using big data. ML could potentially produce a highly reliable diabetes prediction model, a helpful tool for improving lifestyle and preventing diabetes.

## Introduction

Diabetes is a very common disease. It is estimated that 536.6 million people in the world have diabetes, and worldwide diabetes-related health expenditure reached approximately USD 966 billion in 2021^1^. Diabetes leads to severe diseases such as retinopathy, neuropathy, and nephropathy, which decrease the quality of life and can lead to death^2^. Type 2 diabetes is caused by lifestyle habits and can be prevented by efforts made by individuals if they know the diabetes risks they are exposed to and change their lifestyles accordingly^3^. To inform individuals about their risk for diabetes and promote preventive behaviors, it is necessary to predict the future risk of diabetes. When developing such risk prediction models, the estimated risk must be as accurate as possible^4^. While several papers pointed out that many studies did not assess model reliability^4–6^, the importance of reliability has increasingly been recognized^7^. Here, reliability is the measure of the agreement between the predicted probability from the model and observed outcomes^8^; for example, suppose a diabetes prediction model predicts a 10% risk for 100 people, and 10 out of the 100 people develop diabetes in the future. In this case, we regard it as a highly reliable model.

Various models have been developed to predict diabetes risk, and classical statistical models such as logistic regression (LR) are commonly used^4–6,9^. At the moment, immense healthcare-related data are available—data on lifestyle-related diseases, such as diabetes, are actively collected^10–12^. Therefore, efficient and scalable machine learning (ML) models are becoming popular in taking advantage of such big data^11–15^. Among various ML models, the gradient boosting decision tree (GBDT) model^16^ has been found to be highly effective in numerous tasks^17,18^, as its efficient implementation has recently been released^19,20^.

In recent years, however, several papers on clinical prediction models have reported that the performance of ML is rarely different from that of LR. Christodoulou et al. concluded that ML had no performance benefit over LR for clinical prediction models from the meta-analysis in his systematic review^21^. Some studies on the development of diabetes prediction models also reported that ML was as good as LR^22,23^. However, there are two problems with the studies comparing ML and LR. First, they have rarely reported the reliability of prediction models. Silva et al. reported that many studies had evaluated these models for discrimination performance; however, very few studies had evaluated their reliability^24^. Second, there is a possibility that the results of their studies were derived from a lack of sample size. Researchers have pointed out that ML algorithms require a large amount of data to perform better than LR^25^; thus, it is also important to confirm the sample size at which the ML algorithms obtain more reliable predictions than LR.

Against this background, the primary objective of this study is to verify the reliability of ML in developing diabetes prediction models by utilizing big data. We used GBDT as the ML model and compared its reliability to LR models. Moreover, we monitored the data size used when GBDT is more reliable than the LR models. Specifically, we compared the reliability of GBDT and LR while changing the sample size for training. This study was possible due to the big data of health checkups that include 0.6 million individuals every year.

The rest of this paper is organized as follows: In section “Methods”, we describe the big data of health checkups and the cleaning method used to arrive at the effective sample data. We also describe the tendency of the sample data following the training dataset. Further, we describe the two models using the LR and GBDT methods, the reliability diagram, and the evaluation metrics. In section “Results”, we present the results of the reliability diagrams and evaluation metrics for the two methods when changing the sample size. In section “Discussion”, we summarize our findings and mention future perspectives for the study.

## Methods

The Kokuho-database (KDB) consists of big data of the National Health Insurance (NHI) and Senior Elderly Insurance (SEI) in the Osaka prefecture, Japan. The prefecture has approximately 8 million inhabitants, of which approximately 2 million insured individuals are included in the KDB every year. The KDB includes health checkup data, medical receipt data, care receipt data, and their related details for six fiscal years 2012–2017. Our study protocol was approved by the Ethics Committee of Health and Counseling Center, Osaka University (IRB Approval Number 2018–9) and Osaka University Hospital (IRB Approval Number 19073). All procedures involving human participants were conducted per the 1964 Declaration of Helsinki and its later amendments or comparable ethical standards. Informed consent was not obtained from participants because all data were anonymized according to the Japanese Ethical Guidelines for Medical and Health Research Involving Human Subjects enacted by the Ministry of Health, Labor, and Welfare of Japan (https://www.mhlw.go.jp/file/06-Seisakujouhou-10600000-Daijinkanboukouseikagakuka/0000080278.pdf; https://www.mhlw.go.jp/file/06-Seisakujouhou-10600000-Daijinkanboukouseikagakuka/0000153339.pdf).

### Participants

The subjects of this study are insured individuals in the Osaka KDB who received health checkups. We have approximately 0.6 million health checkup data (about 30% of the insured individuals included in KDB), which are recorded in the KDB database every year, for 6 years. We only considered health checkup data from April 2013 to December 2014; thus, we obtained data from 805,816 participants. Among these data, we excluded data of participants where there were inconsistencies in sex or birthday (N = 7). Given that we are using subsequent health checkup data for the outcome of diabetes, we removed those who did not receive health checkups within 3 years of the baseline health checkups—this resulted in clear data from 413,611 participants. We also removed those who had a medical history of diabetes (based on self-reports mentioning that they were receiving treatment for diabetes or were diagnosed with diabetes at a baseline health checkup), or who lacked this information (N = 94,209). As a result, we were left with data from 319,402 participants. The flowchart of the selection of participants is shown in Supplementary Fig. 1.

### Data cleaning

We had to select the variables for developing diabetes prediction models. There were more than 100 items in the health checkup data—including body measurements, blood pressure tests, blood and urine tests, and questionnaires. Of these health indices, we selected items to be used in the analysis by considering the missing rates of the variables (Supplementary Fig. 2). We removed variables with missing rates exceeding 10% to keep the sample size as large as possible. Further, diastolic blood pressure (DBP), aspartate aminotransferase (AST), $\gamma$-glutamyl transpeptidase ($\gamma$-GTP), and urinary glucose (UG) were removed to reduce the effect of multicollinearity. As a result, we decided to use body mass index (BMI) calculated from height and weight, systolic blood pressure (SBP), triglyceride cholesterol (TG), high-density lipoprotein cholesterol (HDL-C), low-density lipoprotein cholesterol (LDL-C), alanine aminotransferase (ALT), glycated hemoglobin A1c (HbA1c), and age as continuous variables. Regarding the categorical variables; we used sex, smoking, ingestion of anti-hypertension (anti-HTN) drugs, ingestion of anti-dyslipidemia (anti-DLP) drugs, urinary protein (UP), medical history (MH) of heart disease, MH of stroke, and MH of renal failure. As for the UP, the measurements were assigned as −, ±, 1+, 2+, and 3+. In this study, we used UP in two classes–these are defined as “negative” for − and ± assignments, and “positive” for 1+, 2+, and 3+ assignments. In the final step, we excluded participants who had missing values, abnormal values (e.g., 0, 999.9), and outliers, which are defined as the outer 0.05% at the base of all distributions at both ends. As a result, 277,651 participants who received health checkups were included in the analysis (Supplementary Fig. 1).

### Outcome

The incidence of diabetes was ascertained using the data obtained from annual health checkups after the baseline health checkup within 3 years. The onset of diabetes was identified following the diagnostic conditions determined by the Japan Diabetes Society^26^—these include the fasting plasma glucose (FPG) of $\ge$ 126 mg/dL, HbA1c of $\ge$ 6.5%, or the self-reported anti-diabetic drugs treatment in the health checkup data. Among the 277,651 participants, 15,900 were newly diagnosed with diabetes within 3 years.

### Prediction models

This section explains the prediction models used in this study. We considered the problem of estimating the conditional probability to create models for predicting the risk of developing diabetes. Let the input space be ${\mathscr {X}}$, and the output space be ${\mathscr {Y}} = \{0, 1\}$. The number of input variables is *d*, which corresponds to the dimension of the input space ${\mathscr {X}}$. We assume that the training data ${\mathscr {D}}_{\text {train}} = \{(\vec {x}_i, y_i)\}_{i=1}^{N}$ are generated independently from the same (unknown) joint probability distribution $p(\vec {x}, y)$. We built models to estimate the conditional probability $p(y=1\mid \vec {x})$ using the training data. We used the LR and GBDT models as the prediction models.

#### Logistic regression model

LR models are used often in the field of epidemiology. They use the following logistic function,

1 $$\begin{aligned} \sigma (x) = \frac{1}{1+e^{-x}}. \end{aligned}$$

Here, the parameters of the LR model are the weight $\vec {w}$ and the intercept *b*, which can be related as $\vec {\theta } = (\vec {w}, b)$. We denote the input variable as $\vec {x}$, and the LR model $f_ {\text {LR}}(\vec {x};\vec {\theta })$ is expressed by the following formula:

2 $$\begin{aligned} f_{\text {LR}}(\vec {x};\vec {\theta }) = \sigma (\vec {w} \cdot \vec {x} + b). \end{aligned}$$

The number of parameters in the logistic model is $d+1$, where *d* stands for the weight of $\vec {w}$ and 1 for the intercept. We fit the parameters of the logistic regression $\vec {\theta }$ by the maximum likelihood estimation. The output probability of the logistic model is $p_i = f_{\text {LR}} (\vec {x}_i; \vec {\theta })$. The maximization of the likelihood corresponds to the minimization of the negative logarithm of the likelihood (NLL), defined as:

3 $$\begin{aligned} \text {Logloss} = - \frac{1}{N} \sum _{i=1}^{N} [y_i \log p_i + (1-y_i) \log (1- p_i)]. \end{aligned}$$

We refer to the NLL as the Logloss function used often in ML. In the use of LR, we first standardized the continuous variables in the training data with a mean of zero and a standard deviation of one. Then, we used the same scaling parameters to standardize the test data.

#### Gradient Boosting Decision Tree

GBDT is an ML algorithm that is widely used due to its effectiveness. It is an ensemble learning algorithm because it learns while adding weak learners additively so that the loss function decreases gradually. The GBDT method uses the decision tree $T(\vec {x};\vec {\theta })$ as weak learners.

4 $$\begin{aligned} T(\vec {x}; \vec {\theta }) = \sum _{j=1}^{J} \gamma _{j} I(\vec {x} \in R_{j}). \end{aligned}$$

Here, *J* is the number of leaves, which are defined by the disjoint regions $R_{j}$ numbered by *j*, and $\gamma _j$ are the values in each region. $\vec {\theta }$ denotes a set of parameters of the decision tree, $\vec {\theta } = (\{\gamma _j\}_{j=1}^J, \{R_j\}_{j=1}^J)$. $I(\vec {x} \in R)$ is the indicator function for the region *R* defined as:

5 $$\begin{aligned} I(\vec {x} \in R) = {\left\{ \begin{array}{ll} 1 &{}\quad (\vec {x} \in R) \\ 0 &{}\quad (\vec {x} \notin R) \end{array}\right. }. \end{aligned}$$

The GBDT model consists of *M* decision trees with parameters $\vec {\theta } = (\vec {\theta }_1, \ldots , \vec {\theta }_M)$. Hence, the GBDT model is written as:

6 $$\begin{aligned} g(\vec {x};\vec {\theta })&= \sum _{m=1}^{M} T(\vec {x}; \vec {\theta }_m), \end{aligned}$$

7 $$\begin{aligned} f_{\text {GBDT}}(\vec {x};\vec {\theta })&=\sigma (g(\vec {x};\vec {\theta })). \end{aligned}$$

We fit the GBDT parameters $\vec \theta$ using the maximization of the likelihood, which corresponds to the minimization of the Logloss function (3). There are various algorithms that can optimize the parameters of the GBDT model. Originally, Friedman proposed the GBDT as a method using the gradient of the loss function^16^. Later, extreme gradient boosting (XGBoost) was proposed using the gradient and Hessian^19^. Ke et al. implemented the light gradient boosting machine (LightGBM), which is an improved version of XGBoost focused on computational efficiency^20^. We adopted LightGBM in our ML algorithm because of its fast training speed. The GBDT model can deal with complex data by increasing the number of parameters; however, this may cause an overfitting problem. Hence, there are various hyperparameters to avoid overfitting. We tuned the hyperparameters of LightGBM with Optuna^27^. Optuna is a Bayesian optimization framework for efficient parameter tuning, and it offers a specialized module for the LightGBM package in Python. The Bayesian optimization approach determines the next parameters to explore based on the history of previously computed parameters. The following parameters were tuned with Optuna according to the default settings: max number of leaves, feature fraction, bagging fraction, bagging frequency, minimal amount of data in one leaf, and L1 and L2 regularization parameters. Further, to avoid overfitting, we performed early stopping when the Logloss of the validation set did not improve for 30 iterations continuously during the training process. We performed hyperparameter optimization for LightGBM using the stratified k-fold cross-validation with k=5.

### Reliability diagram

We introduced the reliability diagram to graphically estimate the goodness of the predicted probabilities^28,29^. We use the test data ${\mathscr {D}}_{\text {test}} = \{(\vec {x}_i, y_i)\}_{i=1}^N$ for the evaluation and describe the quantities to be plotted in the reliability diagram. We first define the trained model as $f : {\mathscr {X}} \rightarrow [0,\, 1]$, and the predicted probability $p_i = f(\vec {x}_i)$ with the input variable $\vec {x}_i \, (i=1,\ldots ,N)$. We then divide the probability $[0,\, 1]$ into *M* equal interval disjoint regions $B_m$, where $m=1,\ldots , M$. Let $I_m$ be a set of subscripts with the predicted probability $p_i$ in the area $B_m$.

8 $$\begin{aligned} I_m = \{ i \in {\mathscr {N}} \mid p_i \in B_m\}, \end{aligned}$$

where ${\mathscr {N}}$ is a set of subscripts for all test data, which is ${\mathscr {N}} = \{1,\ldots , N\}$. The number of data points in each area $B_m$ is written as $N_m$. The average of the predicted probabilities of each bin is:

9 $$\begin{aligned} \bar{p}_m = \frac{1}{N_m} \sum _{i\in I_m} p_i. \end{aligned}$$

Given that $I_m$ fixes the samples in the area $B_m$, we can calculate the expectation value $\bar{y}_m$ of $y_i$ in each region *m*,

10 $$\begin{aligned} \bar{y}_m = \frac{1}{N_m} \sum _{i\in I_m} y_i. \end{aligned}$$

In the reliability diagram, we plot $\bar{p}_m$ in area $B_m$ in the *x*-axis, and $\bar{y}_m$ in the *y*-axis. The reliability of the model is observed graphically by determining how close the reliability diagram is to the diagonal line (45$^\circ$ line).

### Evaluation metrics

Given that the reliability diagram is a graphical representation of the reliability of the model predictions, we needed to introduce some metrics for quantitative evaluation. To this end, we used the expected calibration error (ECE) to quantitatively evaluate the reliability of the probabilities of the model prediction. ECE is the expectation value of the absolute value of the difference between the probabilities of the model prediction and the actual data for various regions.

11 $$\begin{aligned} \text {ECE}&= \sum _{m=1}^M \frac{N_m}{N} |\bar{y}_m - \bar{p}_m|. \end{aligned}$$

Here, *M* is the number of regions in the predicted probabilities. We also used the Logloss function as the evaluation metric, which is defined in Eq. (3). The area under the curve (AUC) of the receiver operating characteristic (ROC) was analyzed for discrimination ability.

### Model performance

To evaluate the model performance, we adopted the *k*-fold cross-validation method for both methods with $k=5$. First, we divided the data into five groups; then, we picked one of these groups as the test data, with the remaining four groups being used as the training data in the first round. When splitting data into training and testing sets, we used stratified random sampling to preserve the proportion of positive cases. We constructed the prediction model using the training data. Then, we ran the prediction model with the test data to calculate ECE, Logloss, and AUC. We repeated this procedure five times to obtain five models and calculated the mean and standard deviation of the evaluation metrics.

### Sample size analysis

We further evaluated the sample size effect of each model on the reliability of the predicted probabilities. To this end, we developed models with LR and LightGBM algorithms using the training data with various sample sizes. Then, we evaluated the reliability using the common test data. We used the reliability diagram and the Logloss and ECE metrics for reliability evaluation. We also calculated AUC for the evaluation of the discrimination. Specifically, we divided the original data into training and test datasets; 80% of the original data was used as training data, with the remaining 20% used as test data. We obtained training data of various sizes by performing random sampling without replacement on the entire training dataset. We varied the size of training data between 1000 and 100,000. Further, we sampled the training data 100 times to calculate the mean and standard deviation of the evaluation metrics.

## Results

### Participant characteristics

**Figure 1:**
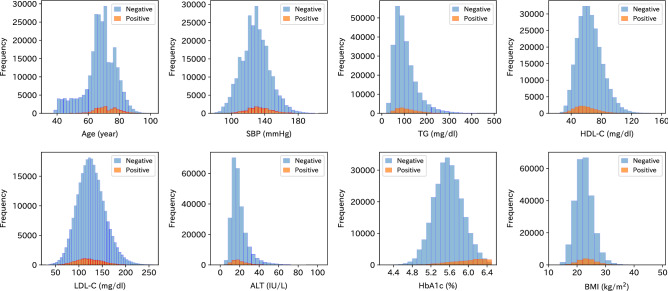
Distributions of all the continuous variables. The histograms of the negative group are denoted by a blue color, whereas those of the positive group are denoted by an orange color. The distributions are truncated at 100 for ALT and 500 for TG for better presentation.

We used the checkup data of 277,651 participants. Among the participants, 15,900 (7978 males and 7922 females) were diagnosed with diabetes in the follow-up checkups. Table 1 shows the characteristics of the variables of the checkup data, where those newly diagnosed with diabetes within 3 years are in the positive group, and those not diagnosed with diabetes are in the negative group. The proportion of females in the positive group was lower than that in the negative group, and age and BMI were higher in the positive group. Moreover, SBP, TG, ALT, and HbA1c were higher, and HDL-C and LDL-C were lower in the positive group. The proportions of those smoking, taking medicines, and with medical histories were higher in the positive group. The positive group also had a higher rate of positive UP. We also show the distributions of the continuous variables for positive and negative groups in Fig. 1.

**Table 1:** Characteristics of variables for all subjects and those in the positive and negative groups. Values are presented as median [Q1, Q3] for continuous variables, and the number *n* and percentage in brackets for categorical variables. *BMI* body mass index, *SBP* systolic blood pressure, *TG* triglyceride cholesterol, *HDL-C* high-density lipoprotein cholesterol, *LDL-C* low-density lipoprotein cholesterol, *ALT* alanine aminotransferase, *HbA1c* glycated hemoglobin A1c, *UP* urinary protein, *HTN* hypertension, *DLP* dyslipidemia, *MH* medical history.

	All (N = 277,651)	Negative (N = 261,751)	Positive (N = 15,900)
Age (years)	68.0 [63.0, 75.0]	68.0 [63.0, 74.0]	71.0 [66.0, 77.0]
BMI (kg/m$^2$)	22.3 [20.4, 24.4]	22.3 [20.4, 24.3]	23.5 [21.4, 25.7]
SBP (mmHg)	128.0 [118.0, 139.0]	128.0 [117.0, 138.0]	132.0 [122.0, 142.0]
TG (mg/dL)	93.0 [69.0, 128.0]	92.0 [68.0, 127.0]	107.0 [79.0, 149.0]
HDL-C (mg/dL)	63.0 [52.0, 73.0]	63.0 [53.0, 74.0]	57.0 [48.0, 68.0]
LDL-C (mg/dL)	124.0 [105.0, 145.0]	125.0 [106.0, 145.0]	121.0 [102.0, 143.0]
ALT (IU/L)	17.0 [13.0, 22.0]	17.0 [13.0, 22.0]	19.0 [14.0, 26.0]
HbA1c (%)	5.5 [5.3, 5.8]	5.5 [5.3, 5.7]	6.0 [5.7, 6.2]
Female	169,779 (61.1)	161,857 (61.8)	7922 (49.8)
Smoking	29,998 (10.8)	28,010 (10.7)	1988 (12.5)
UP			
Negative	262,955 (94.7)	248,453 (94.9)	14,502 (91.4)
Positive	14,696 (5.3)	13,298 (5.1)	1398 (8.8)
Anti-HTN drug	99,612 (35.9)	91,230 (34.9)	8382 (52.7)
Anti-DLP drug	67,923 (24.5)	62,747 (24.0)	5176 (32.6)
MH stroke	9605 (3.5)	8733 (3.3)	872 (5.5)
MH heart disease	16,462 (5.9)	14,972 (5.7)	1490 (9.4)
MH renal failure	1043 (0.4)	954 (0.4)	89 (0.6)

### Model performance

**Figure 2:**
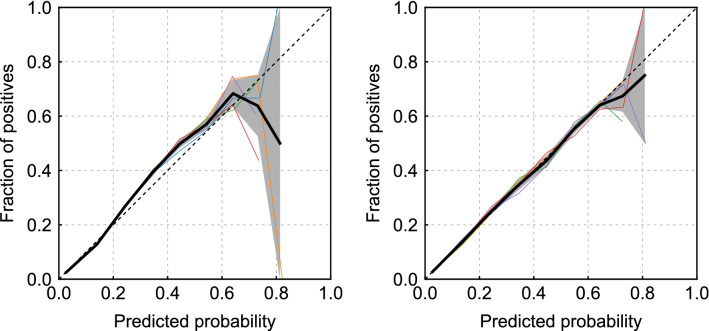
Reliability diagrams for the LR model (left) and LightGBM (right). The horizontal and vertical axes are the predicted probabilities and fraction of positives. There are five curves owing to the 5-fold cross-validation method. The thick black line is the mean of five curves, and the gray area represents the standard deviation.

The prediction reliability of every model was measured by the reliability diagram, which is a visual representation of the model performance for predicted probability. Figure 2 shows the reliability diagrams for the LR and LightGBM prediction models. As can be seen, the reliability curve of the LR model lies almost on the diagonal line except for the portion around 20–50%, where the calculated percentages slightly underestimated the rates of the test data. Meanwhile, when the prediction probability is less than 60%, the reliability curve of LightGBM lies on the diagonal line, and there is no underestimation as in LR. Their reliabilities were also measured based on ECE and Logloss. The LR model achieved an ECE of 0.0048 ± 0.00058 (mean ± standard deviation), and a Logloss of 0.172 ± 0.00090. The LightGBM model achieved an ECE of 0.0018 ± 0.00033 and a Logloss of 0.167 ± 0.00062. Their discrimination performances were measured in AUC, where LR achieved an AUC of 0.826 ± 0.0035, and LightGBM achieved an AUC of 0.844 ± 0.0025.

### Sample size analysis

**Figure 3:**
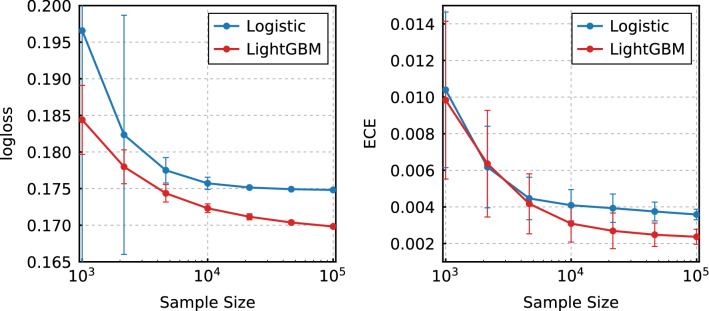
Model performances of the LR and LightGBM models with various sample sizes. The figure on the left shows the Logloss values as functions of the sample size, and the figure on the right shows the ECE values. The error bars are the standard deviation of 100 trials.

Now, to determine the effect of sample size on performance, we evaluated the reliabilities with various sample sizes by randomly compiling the data into small datasets. The reliability was measured by the ECE and Logloss metrics, and these values are shown in Fig. 3 for the LR and LightGBM methods. The corresponding table is presented in the Supplementary Table 1. As can be seen, the Logloss value of the LR model improves up to the sample size of approximately $10^4$, after which the value tends to be saturated as seen in Fig. 3. Meanwhile, the Logloss value for LightGBM continues to improve as the sample sizes increase. Similarly, the ECE indicated that the performance of the two models was similar up until approximately 4500 samples. Further, the ECE tended to be smaller for LightGBM at $10^4$ samples or more. This finding indicates that LightGBM becomes better than the LR method as the sample size increases to more than $10^4$.

**Figure 4:**
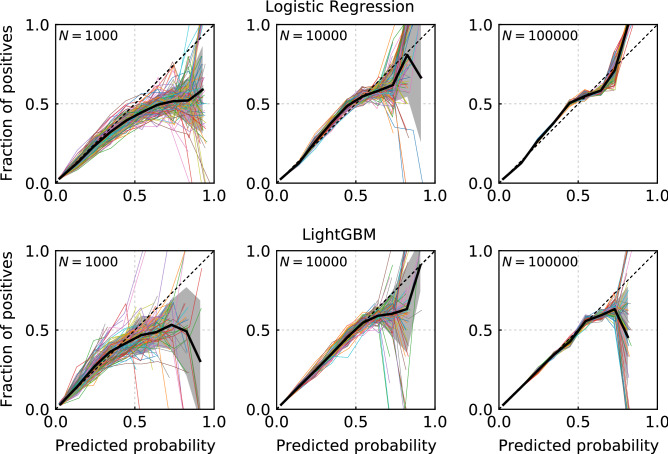
Reliability diagrams for the LR (upper figures) and LightGBM (lower figures) methods with various sample sizes. The samples of $N=1000$, $N=10{,}000$, and $N=100{,}000$ are shown in the left, middle, and right figures, respectively. There are 100 reliability curves in each figure.

The reliability curves for various sample sizes are shown in Fig. 4. There are 100 reliability curves in each figure. When the sample size for training is 1000, the reliability curves scatter largely and tend to distribute under the diagonal line. We note that LightGBM results scatter more largely than those of the LR method. As the sample size increases to 10,000, the reliability curves tend to lie on the diagonal line until the predicted probability is approximately 50%. At a sample size of 100,000, the reliability curves do not scatter largely up to approximately 60%. The reliability curves tend to lie on the diagonal line for the case of LightGBM. Meanwhile, the reliability curves tend to deviate to the upper side of the diagonal line in the probability range of 20–50% for the case of the LR method.

**Figure 5:**
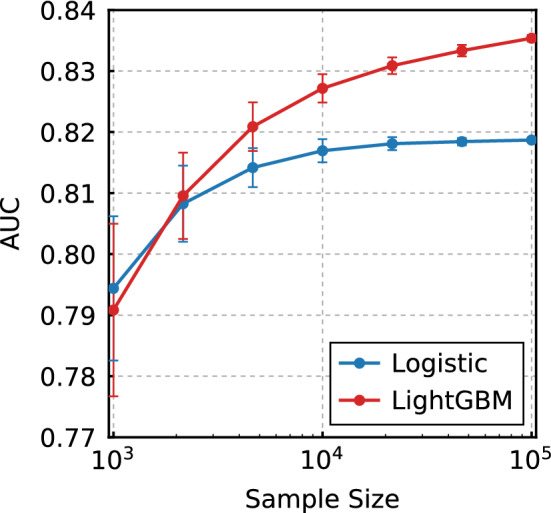
AUC values with various sample sizes for the LR and LightGBM methods.

We also obtained AUC values for various sample sizes. The results are shown in Fig. 5. The AUC values for the LR and LightGBM methods are shown as functions of the sample size. When the sample size is 10,000 or more, LightGBM has better discrimination performance than LR, and the difference becomes larger as the sample size increases.

## Discussion

We developed prediction models for predicting diabetes diagnosis using LR and LightGBM algorithms and compared their reliabilities to identify the algorithms that can utilize big data effectively. Previously, it had been noted that calibration evaluation tends to be omitted in the development of predictive models using ML, even though it is important for evaluating the predictive quality for individuals^5,21,30^. In a systematic review of diabetes prediction models using ML, nine studies compared complex ML and LR^24^; however, only Wang et al. referred to the calibration ability^31^. Moreover, their study only performed the Hosmer–Lemeshow test and did not quantitatively compare the reliability of the models. Notably, the Hosmer–Lemeshow test has the shortcoming that it is nearly always statistically significant for large sample sizes^5^. Indeed, Paul et al. do not recommend using the Hosmer–Lemeshow test when the sample size is greater than 25,000^32^. In these situations, when comparing the two algorithms with the total amount of data, we found that the reliability of LightGBM was better than that of LR. Further, the reliability of the two models was evaluated with various training data sizes to determine the sample size at which complex ML models are effective for big data. When the sample size exceeded 10,000, LightGBM outperformed LR in the two quantitative metrics of reliability. We also observed that, at over 10,000 participants, LightGBM showed a greater rate of improvement with sample size in Logloss compared to LR.

The difference between the LR and LightGBM algorithms arises from whether the function inserted into the sigmoid function is linear or nonlinear. In LR, this function can only assume a linear hyperplane in the input variable space. In LightGBM, the function can assume a high flexibility distribution. We can understand these results by analyzing Eq. (2), where the LR method has only “the number of input variables + 1” as the total number of parameters, and in the present case, only 17 parameters. Therefore, the LR model has a limited ability to adapt data to the model, and there is a possibility that this ability is limited to approximately 10,000 participants. Meanwhile, the LightGBM method can increase the number of parameters without limit as long as it can learn, as explained in the GBDT method. Therefore, as the sample size increases, it is conceivable that the performance improves due to the flexibility of GBDT as it allows for an increased number of parameters. Although LightGBM tends to overfit as the number of parameters increases, we avoided overfitting by setting the appropriate hyperparameters, such as the number of trees, number of leaves, and regularization parameters. Thus, the calibration in our study was improved as the amount of data increased.

We also observed that LightGBM showed a better score and greater improvement in AUC than LR when the sample size exceeded 10,000. This result may imply that the results of some previous papers concluding that there is no difference in performance between modern ML algorithms and classical linear algorithms may be due to their small sample size. For example, to compare the linear regression model and LightGBM, Kopitar et al. calculated AUC values for discrimination of diabetes^22^. They reported that the linear regression model outperforms LightGBM in the AUC values in all datasets. In their study, the data used to construct the model was always less than 3000. Furthermore, Christodoulou et al. compared 13 articles with a low risk of bias and concluded that, on average, there are no significant differences in the AUC performances of LR and ML models^21^. However, in the 13 articles, ML tended to outperform LR when the training data size was over 10,000.

In our study, the number of people diagnosed with diabetes within 3 years is only 5.7%. In our unbalanced data, the closer the prediction observation probability is to 1, the smaller is number of data. Therefore, the errors become large at higher observation probability areas. Further, the ECE value is weighted by the ratio of the amount of data in each bin to the total amount of data. Thus, the contribution of ECE from higher observation probability areas is small. Recognizing these problems, we will consider a more suitable reliability metric for the evaluation of unbalanced data in the future.

Other limitations of our study include the following: (1) the NHI and SEI datasets could be merged, and therefore, the participants who moved from NHI to SEI for the follow-up checks needed to be excluded from our study, which, in turn, led to the exclusion of many participants aged between 72 and 75 years from our datasets. (2) The ages at the baseline health checkups are not accurate. By anonymizing the personal information, our datasets only provided the date of the health checkup, and the year of birth. As the age was calculated from this information, the age values have an error of at most 1 year.

## Conclusion

We confirmed that GBDT provides a more reliable model than LR when the training sample size exceeds 10,000 in the development of diabetes prediction models using big data. This result indicates that ML may be an effective prediction model development tool in the era of big healthcare data. Specifically, ML could potentially produce a highly reliable diabetes prediction model, which is a helpful tool for improving lifestyle to prevent diabetes.

In this study, only the LightGBM method was used as the ML model. In the future, we will perform sample size analysis on many other ML models, such as artificial neural networks, and support vector machine. Moreover, we will investigate the cause of diabetes by using the technologies, such as the partial dependence plot^16^ and SHAP (SHapley Additive exPlanations)^33^, in ML. Furthermore, it is necessary to investigate the effects of predicting diabetes on individual risk perception and behavior. Considering the results obtained in this study, in the future, we will investigate these factors from the viewpoint of promoting appropriate behavior change according to risks.

## Supplementary Information

Supplementary Information.

## Data Availability

Data cannot be shared publicly because local governments own medical check-up data. Data are available from the Health and Counseling Center, Osaka University (contact via campuslifekenkou-syomu@hacc.osaka-u.ac.jp) for researchers who meet the criteria for access to confidential data.

## Code availability

One of the GBDT-based diabetes prediction models used for evaluating the model performance is available on GitHub (https://github.com/AsukaOyama/GBDT-DM-Prediction) and Zenodo (10.5281/zenodo.7024546).
