# Author Correction: Gradient boosting decision tree becomes more reliable than logistic regression in predicting probability for diabetes with big data

**DOI:** 10.1038/s41598-022-27052-7

**Published:** 2022-12-30

**Authors:** Hiroe Seto, Asuka Oyama, Shuji Kitora, Hiroshi Toki, Ryohei Yamamoto, Jun’ichi Kotoku, Akihiro Haga, Maki Shinzawa, Miyae Yamakawa, Sakiko Fukui, Toshiki Moriyama

**Affiliations:** 1 Health Care Division, Health and Counseling Center, Osaka University, Osaka, 560-0043 Japan; 2 Graduate School of Human Sciences, Osaka University, Osaka, 565-0871 Japan; 3 Research Center for Nuclear Physics, Osaka University, Osaka, 567-0047 Japan; 4 Department of Nephrology, Graduate School of Medicine, Osaka University, Osaka, 565-0871 Japan; 5 Health Promotion and Regulation, Department of Health Promotion Medicine, Osaka University Graduate School of Medicine, Osaka, 565-0871 Japan; 6 Graduate School of Medical Care and Technology, Teikyo University, Tokyo, 173-8605 Japan; 7 Graduate School of Biomedical Sciences, Tokushima University, Tokushima, 770-8503 Japan; 8 Division of Health Sciences, Graduate School of Medicine, Osaka University, Osaka, 565-0871 Japan; 9 Department of Home and Palliative Care Nursing, Graduate School of Health Care Sciences, Tokyo Medical and Dental University, Tokyo, 113-8519 Japan

Correction to: *Scientific Reports* 10.1038/s41598-022-20149-z, published online 11 October 2022

The original version of this Article contained an error in Affiliation 2, which was incorrectly given as ‘Department of Human Science, Osaka University, Osaka 560-0043, Japan’.

The correct affiliation is listed below:

Graduate School of Human Sciences, Osaka University, Osaka 565-0871, Japan

The original Article and accompanying Supplementary Information file have been corrected.

