# Supplementary material for: Gradient boosting decision tree becomes more reliable than logistic regression in predicting probability for diabetes with big data
Source: Sci Rep. 2022 Oct 11;12:15889. doi: 10.1038/s41598-022-20149-z (PMC9553945; doi:10.1038/s41598-022-20149-z)
Supplement: Supplementary file 1 — Supplementary Information. [file 41598_2022_20149_MOESM1_ESM.pdf]

Supplemental material to the paper on "Gradient Boosting Decision Tree Becomes More Reliable Than Logistic Regression in Predicting Probability for Diabetes With Big Data"

Hiroe Seto[1,2], Asuka Oyama*[1], Shuji Kitora[1], Hiroshi Toki[1,3], Ryohei Yamamoto[1,4,5], Jun'ichi Kotoku[1,6], Akihiro Haga[1,7], Maki Shinzawa[4], Miyae Yamakawa[8], Sakiko Fukui[8,9], and Toshiki Moriyama[1,4,5]

[1] *Health Care Division, Health and Counseling Center, Osaka University, Osaka 560-0043, Japan*
[2] *Graduate School of Human Sciences, Osaka University, Osaka 565-0871, Japan*
[3] *Research Center for Nuclear Physics, Osaka University, Osaka 567-0047, Japan*
[4] *Department of Nephrology, Graduate School of Medicine, Osaka University, Osaka 565-0871, Japan*
[5] *Health Promotion and Regulation, Department of Health Promotion Medicine, Osaka University Graduate School of Medicine, Osaka 565-0871, Japan*
[6] *Graduate School of Medical Care and Technology, Teikyo University, Tokyo 173-8605, Japan*
[7] *Graduate School of Biomedical Sciences, Tokushima University, Tokushima, 770-8503, Japan*
[8] *Division of Health Sciences, Graduate School of Medicine, Osaka University, Osaka 565-0871, Japan*
[9] *Department of Home and Palliative Care Nursing, Graduate School of Health Care Sciences, Tokyo Medical and Dental University, Tokyo 113-8519, Japan*

Abstract

This is supplementary material to the paper entitled "Gradient Boosting Decision Tree Becomes More Reliable Than Logistic Regression in Predicting Probability for Diabetes With Big Data". Here, we provide several detailed backup pieces of information to assertions in the main text.

1 Data selection and cleaning

The flowchart of the number of participants is shown in supplementary Fig. 1. There were 805,816 individuals in the baseline health checkup data from April 2013 to December 2014. We excluded data of individuals who had inconsistencies in sex or birthday (N=7). Given that we used subsequent checkup data to determine the outcome of diabetes, we also excluded data of individuals who did not receive health checkups within three years of the baseline health checkup (i.e., 413,611 individuals)

Those who had a medical history of diabetes (based on: (1) self-reports that they receiving treatment for diabetes, (2) diagnosed with diabetes at baseline health checkup,

*Correspondence to ooyama@hacc.osaka-u.ac.jp

or (3) absence of the aforementioned two pieces of information) were also removed (N=94,209).

Participants who had missing values, abnormal values (e.g. 0, 999.9), and outliers, which are defined as outer 0.05% at the baseline of all distributions at both ends were also excluded.

As a result, only 277,651 participants remained for analysis. The flowchart of the selection of participants is shown in supplementary Fig. 1, and the percentages of missing values in health indices are shown in supplementary Fig. 2.

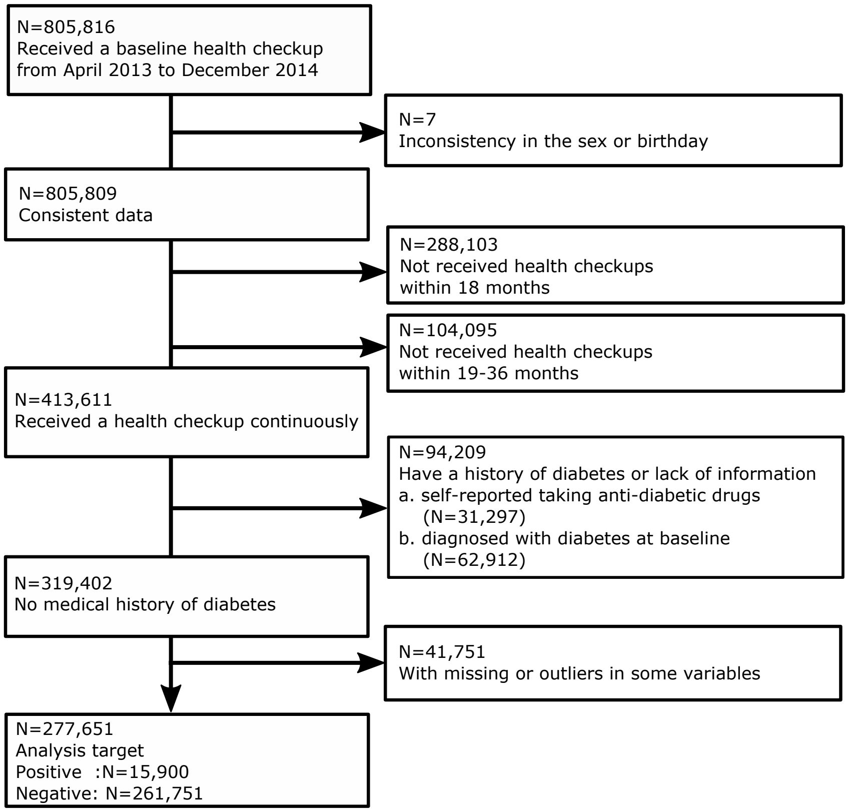

Supplementary figure 1. Flowchart of participants.

2 Evaluation metrics

Here, we provide a table for the evaluation metrics of the LR and LIghtGBM models for various sample sizes. ECE and Logloss metrics were chosen to evaluate model reliability. Supplementary table 1 shows the metrics for the LR model on the left-hand side and those for LightGBM on the right-hand side. These evaluation metrics are shown in Fig. 3 of the main text alongside the related discussions.

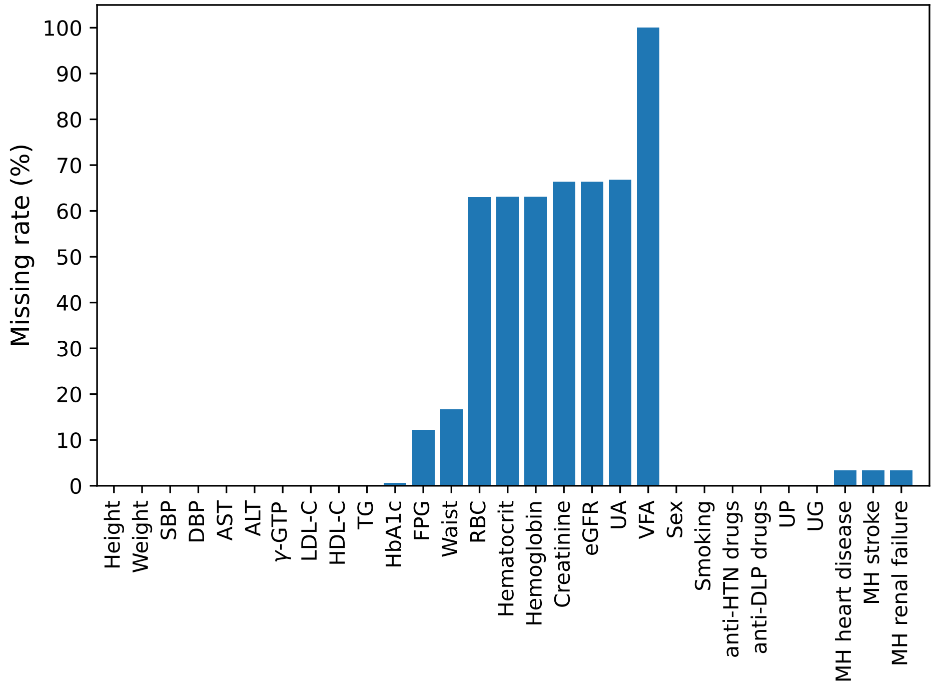

Supplementary figure 2. Health indices and the percentages of missing values. All the abbreviations of the horizontal axis are explained in the main text.

Supplementary table 1. Values of the ECE and Logloss metrics for various sample sizes. Each value for the LR model is shown in the left-hand side and that for LightGBM in the right-hand side. The mean values and their standard deviations of 100 trials are listed in brackets.

	Logistic regression			LightGBM		
Sample size	Logloss	ECE	AUC	Logloss	ECE	AUC
1000	0.197 (0.041)	0.010 (0.004)	0.794 (0.012)	0.184 (0.005)	0.010 (0.004)	0.791 (0.014)
2154	0.182 (0.016)	0.006 (0.002)	0.808 (0.006)	0.178 (0.002)	0.006 (0.003)	0.810 (0.007)
4641	0.178 (0.002)	0.004 (0.001)	0.814 (0.003)	0.174 (0.001)	0.004 (0.002)	0.821 (0.004)
10000	0.176 (0.001)	0.004 (0.001)	0.817 (0.002)	0.172 (0.001)	0.003 (0.001)	0.827 (0.002)
21544	0.175 (0.000)	0.004 (0.001)	0.818 (0.001)	0.171 (0.000)	0.003 (0.001)	0.831 (0.001)
46415	0.175 (0.000)	0.004 (0.001)	0.818 (0.001)	0.170 (0.000)	0.002 (0.001)	0.833 (0.001)
100000	0.175 (0.000)	0.004 (0.000)	0.819 (0.000)	0.170 (0.000)	0.002 (0.000)	0.835 (0.001)
